# Research on Effect of Load Stimulation Change on Heart Rate Variability of Women Volleyball Athletes

**DOI:** 10.1155/2022/3917415

**Published:** 2022-03-19

**Authors:** Ludi Liao, Jianying Li

**Affiliations:** School of Physical Education Shanxi University, Taiyuan, Shanxi 030006, China

## Abstract

*Objective*. To explore the effect of different training load stimulation on heart rate variability level of Chinese elite female volleyball players. Through two-year follow-up experiment, this paper uses OmegaWave Sport Technology system to track and test the heart rate variability level and central nervous system parameters of 25 elite Chinese women volleyball players who participated in the national adult volleyball training in 2019 and 2020. It is found that the HRV time-domain index of the players under the stimulation of three stages of training load during the winter training in 2020 is determined. Frequency-domain index has significant influence on response stability of central nervous system. In order to further explore the influence of HRV on response stability of central nervous system, a feature classification method based on distance evaluation is proposed for experimental data processing. Through the multimodal human-machine interaction (M-HMI), advanced machine learning is used to promote the cooperative interaction between human and intelligent body. After analysis, SDNN and LF n.u. have a significant impact on the average reaction time. It shows that some indexes tested by the OmegaWave system can reflect the real-time physical function state of athletes sensitively and play an active role in diagnosis of fatigue of athletes' central nervous system. HRV time-domain and frequency-domain indexes, as parameters to evaluate the body functional state of excellent female volleyball players in the preparation process of competition, can sensitively reflect the level of autonomic nerve regulation of athletes in three different load stages.

## 1. Introduction

Volleyball, as one of the three major balls, has a wide social impact and bears the spirit of the times of the country and nation. In the 1980s, Chinese women's volleyball team reached the historical peak of “five consecutive crowns.” In the 21st century, Chinese women's volleyball team won the world championship five times and then made great achievements. Adult women's volleyball team is the frontline team of Chinese women's volleyball team and the basis and guarantee for Chinese women's volleyball team to maintain the world-class team. With the increasing competitive level of modern volleyball and the increase of training load and competition pressure, it is very possible to promote sports fatigue of athletes, which will make them unable to fulfill the technical and tactical requirements laid out by coaches in training and competition and ultimately lead to the decline of competition ability and affect the effect of training and competition [1–3]. Therefore, how to quickly and accurately monitor the athlete's body function and the functional level of central nervous system and timely understand the athlete's functional state has become an urgent problem for high-level sports teams [4, 5].

Due to the change of external load, a series of physiological and biochemical changes will inevitably take place in athletes. These changes can be counted and measured by detecting physiological and biochemical related indexes. Among the many physiological indexes for measuring sports load, heart rate variability (HRV) is a method of measuring biofeedback that is receiving increasing attention, mainly due to its high technical availability and portability [6, 7]. Exercise load is positively and negatively related to heart rate variability [8]. Increased exercise load leads to inconsistency of ANS functions, which negatively affects HRV [8, 9]. When an athlete responds to a load intensity, the SNS is activated to allow the athlete to respond appropriately to the sporting needs. However, when an athlete experiences a load stimulus that exceeds his or her current level of acceptance, the SNS response increases [10]. Increased activation of SNS decreases the function of the vagus, which is critical to maximizing and lowering the heart rate during RSA [11]. Therefore, a decrease or even disappearance of vagus nerve tension can reduce the rhythm and have a negative impact on heart rate variability. Therefore, heart rate variability (HRV) is considered by many researchers as a marker of homeostasis [12] and is widely used as an indicator of training adaptability in sports environment [13–17]. Factors such as training load, type, stage, type of competition, and level of health [18–20] have proved to influence HRV level.

In recent years, great advances have been made in computational intelligence and machine learning methods, which have driven the deployment of neural networks and intelligent systems in many life scenarios and industrial fields. Multimodal human-computer interaction system (M-HMI) mainly includes EEG signal and ECG signal. The traditional single-mode human-computer interaction system has been unable to meet the actual needs due to its few task categories, so the multimodal human-computer interaction system came into being. At the same time, other types of interaction are also gradually applied to the human-computer interaction system. Based on this, a multimodal human-machine interaction system based on distance assessment feature classification was constructed by combining ECG signal and EEG signal, which solved the problems of hardware and software platform construction and signal synchronization, and an effective feature classification processing method was proposed.

This study utilizes OmegaWave Sport Technology system. Using omegawave sport technology system, this study analyzes 13 (2019) and 12 (2020) teams participating in China Volleyball Super League. This paper studies the changes of physical function and central nervous system function level of athletes in different intensity training. According to the changes of physiological indexes of athletes after different load training, the experimental results provide a reference for load arrangement of adult women volleyball training course. At the same time, it can improve the training efficiency of women volleyball team. It provides theoretical basis for precompetition training of women volleyball players.

## 2. Proposed Feature Classification Method Based on Distance Evaluation

The goal of cluster analysis is to collect data and classify them on a similar basis. In this paper, XGBoost is used to model and classify the experimental results, and the possible overfitting caused by XGBoost is improved by calculating the coefficient weight of the characteristic through improved distance evaluation method.

XGBoost can adapt itself to learn a certain number of samples with certain characteristics. However, XGBoost's strong learning ability often results in overfitting, which affects the classification results of samples. Therefore, additional conditions are required to limit the learning ability of the XGBoost model. The improved distance evaluation algorithm can calculate the differences between classes of samples to determine the influence of various characteristics in the sample capacity and the size characteristics of the differences between the categories. The improved distance evaluation method is used to obtain the impact weight of sample features in the model tree, so as to improve the overfitting learning of XGBoost and the classification effect of XGBoost.

XGBoost can quickly classify sample characteristics by decision tree classification. Compared with other decision tree models, this model has faster accuracy and calculation speed. Combining the weight between features obtained by improved distance evaluation algorithm with the weight parameters in XGBoost classification tree model can ensure the correlation between data and prevent the overfitting in the XGBoost method. The parameters in the model are calculated as follows.

Enter characteristic dataset, in which the sample category is *Y*, the number of samples in each category is *N*, and the number of features in each sample is *M*:

(1) $$\begin{array}{c} D=\left\{X_{y,n,m},Y\right\}, \end{array}$$

where *X*_*y*,*n*,*m*_ denotes the *n*th sample in the class *y* of a dataset that contains *m* features and *y* is the category of the corresponding sample.

Standard deviation between data within the calculation coefficient:

(2) $$\begin{array}{c} \delta_{y,m}=\sqrt{\frac{\sum_{n=1}^{N}{\left(X_{y,n,m}-u_{y,m}\right)}^{2}}{N-1}}, \end{array}$$

where *u*_*y*,*m*_ represents the average value of the data within the *m*th feature in the dataset:

(3) $$\begin{array}{c} u_{y,m}=\frac{1}{N}\sum_{n=1}^{N}X_{y,n,m}. \end{array}$$

The standard deviation within the coefficient can be obtained:

(4) $$\begin{array}{c} clt_{m}^{\text{inner}}=\frac{1}{Y}\sum_{y=1}^{y}\delta_{y,m}\;. \end{array}$$

There are differences between different categories of data, where *y*, *c* indicate that data belong to different categories:

(5) $$\begin{array}{c} f_{m}^{\text{inner}}=\frac{\max\left(clt_{y,m}^{\text{inner}}\right)}{\min\left(clt_{c,m}^{\text{inner}}\right)}.\; \end{array}$$

Then, the data differences between features are calculated. First, the standard deviation between features is calculated:

(6) $$\begin{array}{c} \tau_{y,m}=\sqrt{\frac{\sum_{i=1}^{M-1}{\left(cd_{n,r,y,m}-d_{y,m}\right)}^{2}}{N\left(N-1\right)-1}},\\ cd_{n,r,y,m}=\left|X_{y,n,m}-X_{y,r,m}\right|,\\ d_{y,m}=\frac{1}{N\left(N-1\right)}\sum_{n,r=1}^{N}cd_{n,r,y,m}.\; \end{array}$$

Calculate the average of standard deviations between features:

(7) $$\begin{array}{c} clt_{m}^{\text{outer}}=\frac{\sum_{y,c=1}^{Y}{\left(\tau_{y,m}-\tau_{c,m}\right)}^{2}}{Y\left(Y-1\right)}. \end{array}$$

Differences between features:

(8) $$\begin{array}{c} f_{m}^{\text{outer}}=\frac{\max\left(clt_{y,m}^{\text{outer}}\right)}{\min\left(clt_{c,m}^{\text{outer}}\right)}\;. \end{array}$$

The distance weight coefficient between the coefficients can be calculated as

(9) $$\begin{array}{c} \eta_{m}=\frac{1}{\left(f_{m}^{\text{inner}}/\text{max}\left(f_{m}^{\text{inner}}\right)\right)+\left(f_{m}^{\text{outer}}/\text{max}\left(f_{m}^{\text{outer}}\right)\right)}\cdot \frac{clt_{m}^{\text{outer}}}{clt_{m}^{\text{inner}}}\;. \end{array}$$

## 3. Numerical Experiments

### 3.1. Experimental Conditions

**Figure 1:**
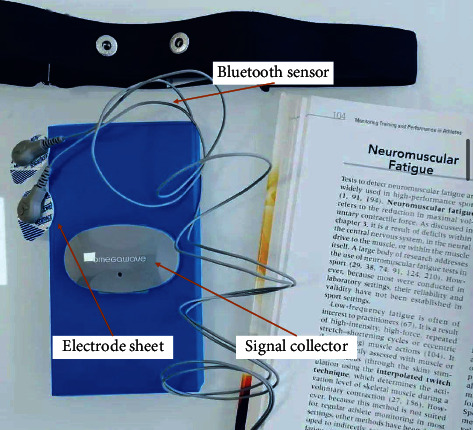
OmegaWave Sport Technology system.

During the test, the OmegaWave Sport Technology system developed by a LLC Corporation was used to sample the athletes at three stages of different training loads during the winter training. The OmegaWave Sport Technology system developed by LLC Corporation of America is used in the test process of this paper. Athletes are sampled at three stages of different training loads during winter training. The TGAM module includes a TGAT chip, which is a highly integrated EEG sensor. It reads human brain signals using dry electrodes, filters out disturbances from ambient noise, and converts the detected brain signals into digital signals. The device automatically detects abnormal contact conditions and filters out electrical noise and 50/60 Hz AC interference. Bluetooth sensor is used to collect EEG information of athletes in different states, and then it is transmitted back through Bluetooth, electrode pieces are used to collect athletes' ECG information, and the signal collector amplifies the collected signal and stores it on the computer after synchronization. The OmegaWave Sport Technology system is shown in Figure 1.

**Figure 2:**
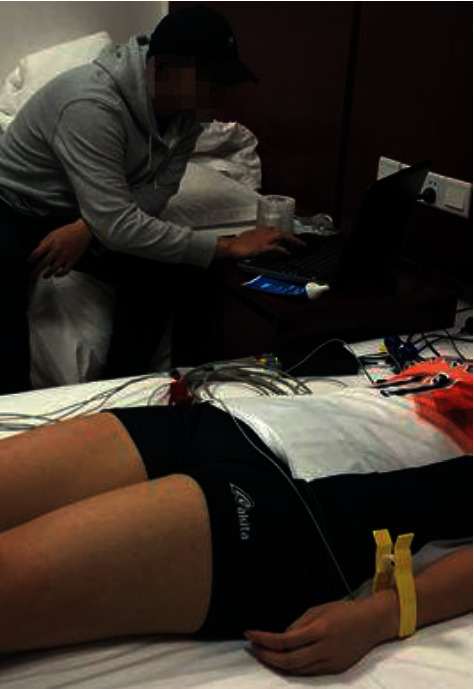
Field data acquisition picture.

The TGAM module can directly connect the dry contact points, unlike the wet sensor used in traditional medicine, which requires conductive adhesive, and the single EEG channel has three contact points: EEG (EEG acquisition point), REF (reference point), and GND (ground point). The collected signal is shown in Figure 2.

The physical quantities were measured as follows:

1. Measuring original brain wave signal.
2. Processing and outputting *α*, *β* isoencephalic band data.
3. Processing and outputting Neurosky's eSense degree of concentration and relaxation index and other data to be developed in the future.

The research object of this study is to study the physical health of 13 (2019) and 12 (2020) teams of Chinese Women's Volleyball Super League in 2019 and 2020 during the training period of National Adult Women's Volleyball Team, each team having 5 athletes, a total of 125 people. The details are shown in Table 1.

This research adopts preexperiment and postexperiment design modes to test the real-time performance of all the tested athletes who participated in the training for two years after the specific training class and to track and monitor the influence of training load arrangement on the HRV level of the athletes at different training stages during the winter training period. Before winter training, the characteristics of training modes and training loads of athletes in three stages, i.e. early stage, middle stage, and late stage of winter training, are classified. The coaches select a training session in this training stage for testing after a training session and the testing time is within one hour after the end of training. Each participant was tested three times during the whole winter training period, during which HRV index data were collected strictly according to the test process.

**Figure 3:**
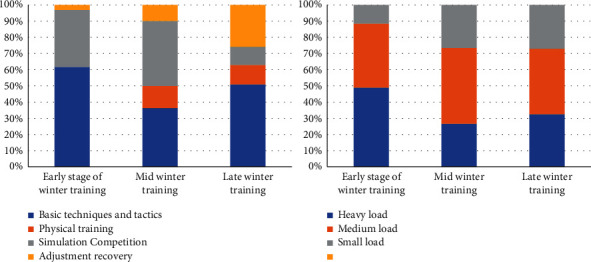
Proportion of winter training course plan and training load in 2019.

**Figure 4:**
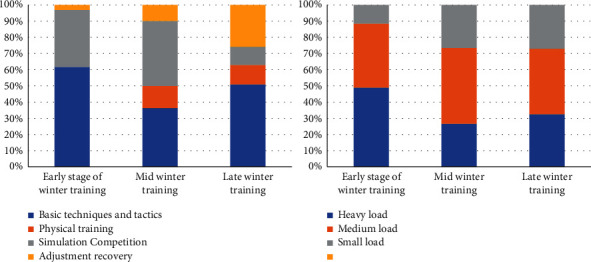
Proportion of winter training course plan and training load in 2020.

As can be seen from Figure 3, in the early stage of winter training in 2019, the basic technical and tactical training is mainly multiball training. Physical training focuses on waist and abdomen strength, lower limb strength, explosive strength, and upper limb strength training. Simulation games mainly focus on group tactical explanation and training. The rest adjustment is based on stretching and active relaxation. In the middle of winter training, the training mode has a certain change, and the basic technical and tactical training is mainly series training. Physical training is mainly based on speed training methods such as step running, slope running, and lateral movement. The simulation competition is mainly based on the actual combat competition of the whole team. The rest adjustment is based on stretching and active relaxation. The latter part of winter training is the combination of the previous two training modes, the basic technical and tactical training to the overall series and confrontation training. Physical training is based on sensitive and flexible training methods. The simulation competition is mainly based on the actual combat competition of the whole team. The training mode for 2020 is shown in Figure 4. The two training modes are the same in structure, but slightly different in time.

### 3.2. Real-Time Performance Testing Process of Athletes

In the OmegaWave real-time functional test, the basic potential at rest has been identified as an indicator of the functional state and adaptive reserve level of the central nervous system. For healthy people, the significance of the fourth-order resting potential is as follows: less than −30 mV—very low level, −29 to −1 mV—low level, 0 to 46 mV—best level, and greater than 47 mV—high level. The basic test procedure is as follows:

1. Enter the basic information such as age, height, weight, and sports grade into OmegaWave system in advance.
2. Check the basic information of athletes and explain the testing process and requirements before starting the test formally.
3. Players lie down in a quiet and comfortable environment.
4. The tester wipes the electrode with an alcohol cotton ball at the position where the electrode is affixed according to the operation requirements and then clips the electrode.
5. Confirm that brain lead, chest lead, and limb lead are connected to computer normally and the athletes have no uncomfortable reaction.
6. Athletes remain relaxed and then begin testing. There are differences in the time of reaching steady state among different athletes and the time of testing. The whole data collection process is completed in about 15 minutes.
7. The test data are collected and the reaction time test is carried out after the relevant lead is removed. The test indexes are shown in Table 2. They mainly include the standard deviation of NN interval (SDNN), the mean square deviation of adjacent NN interval difference (RMSSD), and the standard deviation of adjacent NN interval difference (SDSD).

**Figure 5:**
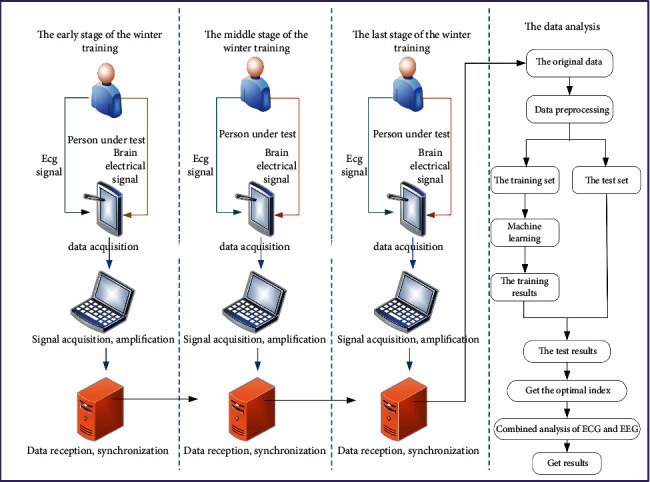
The flowchart of the method.

The testing process is shown in Figure 5. ECG and EEG signals of the tested personnel are collected first, amplified and stored, and then preprocessed for feature classification to obtain correlation coefficients between features.

## 4. Test Results

### 4.1. Variation Characteristics of HRV Time-Domain Indexes of Elite Female Volleyball Players in Different Training Load Stages

Heart rate variability (HRV) index can reflect the activity of the autonomic nervous system, the tension of sympathetic nerve and vagus nerve, and the influence of autonomic nervous system on athletes' heart rate and can reveal the more complex change law of heart rate. There is a certain correlation between exercise load and heart rate variability index. In previous studies, SDNN decreased significantly after exercise, and it is significantly negatively correlated with the increase of biochemical index BLA reflecting load intensity.

**Figure 6:**
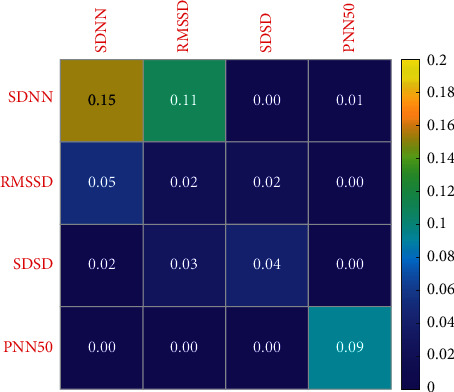
Change sensitivity of HRV time-domain indexes of women volleyball players in different load stages in 2019.

**Figure 7:**
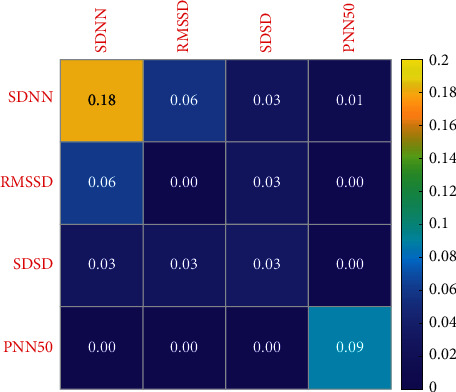
Change sensitivity of HRV time-domain indexes of women volleyball players in different load stages in 2020.

Figures 6 and 7 show the influence of different training load stimulation on HRV time-domain indexes of elite female volleyball players; as can be seen from the figures, SDNN is the most sensitive index, so it can be used to reflect the actual situation of athletes.  Table 3 shows that SDNN of athletes increased gradually with the exercise time. During the winter training in 2020, athletes gradually entered a state of tension, and the ability of autonomic nervous system to regulate heart rate decreased gradually; the change of load intensity and the increase of psychological pressure near the game may be the reasons for this phenomenon. At the same time, from the arrangement of training load in 2020, it can be found that in the test process of the initial stage of winter training, the daily training of volleyball players is mainly based on basic technical training and physical fitness training, the arrangement of simulated competition is less, and the load intensity borne by athletes is also low. In the middle of winter training, targeted training such as attack and defense confrontation has increased; especially, when coaches carry out multigroup attack and defense series training for athletes, the number of touch times, moving distance, and take-off times have increased sharply in a short time, so that the load intensity borne by athletes is greater than that in the early training stage of winter training. In the late winter training period, the athletes' load intensity is also higher and higher. The proportion of simulation competitions is also increasing. This also causes athletes to increase the probability of sports fatigue. Therefore, the proportion of adjustment and recovery is increasing in the arrangement of training plans.

### 4.2. Variation Characteristics of HRV Frequency-Domain Indexes of Elite Female Volleyball Players in Different Training Load Stages

**Figure 8:**
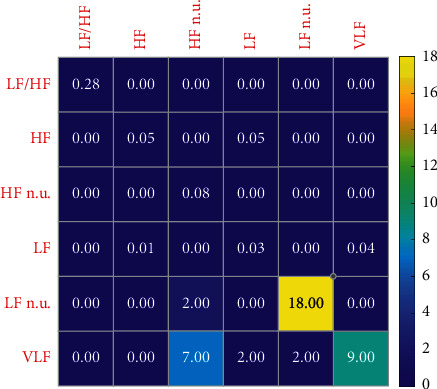
Change sensitivity of HRV frequency-domain index of women volleyball players in different load stages in 2019.

**Figure 9:**
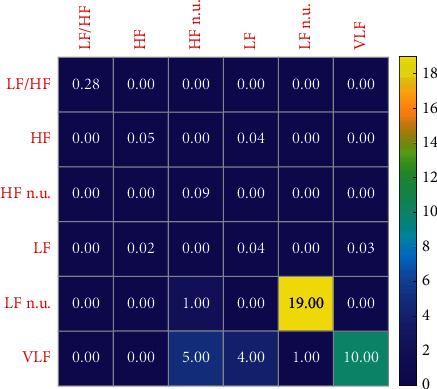
Change sensitivity of HRV frequency-domain index of women volleyball players in different load stages in 2020.

Figures 8 and 9 show the variation characteristics of HRV frequency-domain indicators. As can be seen from the figures, in different training load stages of elite women volleyball players, LF n.u. indicator is the most sensitive and can therefore be used to reflect the actual situation of athletes. As can be seen from Table 4, it first decreases and then increases as the training phase progresses.

This indicates that in the middle stage of winter training, the athletes' vagal tension decreases, their cardiac oxygen consumption increases, and their heart rate recovery slows down after exercise. It may be that the athletes' physical function state at this stage is not fully recovered, or they have not adapted to the exercise load stimulation at this stage. However, in the late stage of winter training, the athletes' training load changes and the proportion of recovery adjustment increases, and their physical function level also recovers. It has exceeded the level of early winter training, or it has not adapted to the exercise load stimulation at the stage, and in the later stage of winter training, with the change of athletes' training load and the increase of recovery adjustment proportion, the level of physical function also recovers, but it does not reach the level at the early stage of winter training. Through the analysis of the proportion of the training plan in the winter training stage in 2020, it can be found that the training focus in the early stage of winter training is mainly on the strengthening basic techniques and tactics. In the middle stage of winter training, the proportion of simulated competitions of each team gradually increases, and the proportion of basic techniques and tactics training and physical training courses gradually decreases. With the winter training reaching the final stage, athletes will have a certain accumulation of sports fatigue and hidden dangers of injuries. In the later stage of winter training, the training plan mainly focuses on simulated competition and recovery adjustment. On the one hand, it tests the effect of technical and tactical training and prepares for future competitions.

### 4.3. Variation Characteristics of Central Nervous System Indexes of Elite Female Volleyball Players in Different Training Load Stages

**Figure 10:**
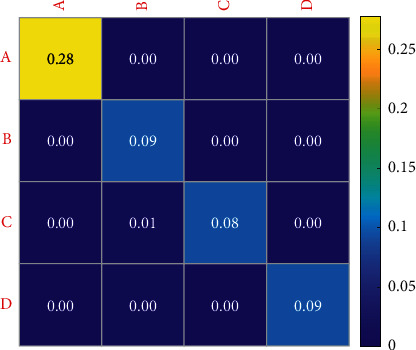
Sensitivity of central nervous system indexes of women volleyball players at different load stages in 2019.

**Figure 11:**
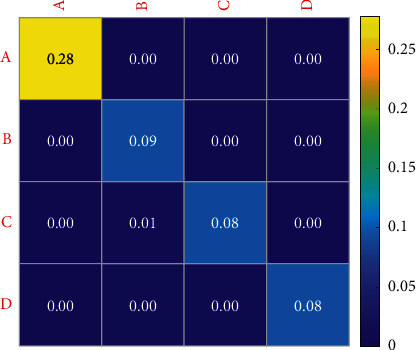
Sensitivity of changes in central nervous system indexes of women volleyball players at different load stages in 2020. A: mean response time; B: sensory motor nervous system function level index; C: response stability index; D: sensorimotor function development potential index.

Figures 10 and 11 show the variation characteristics of HRV frequency-domain indexes of elite female volleyball players at different training load stages. In the test results of this study, the tension index in the later stage of winter training in 2019 is significantly higher than that in the early stage of winter training. As can be seen from Table 5, the reaction time of athletes decreases gradually with training. This phenomenon shows that with the adjustment of winter training load intensity and load, the athletes' stress response to load stimulation reaches the peak in the later stage. In the later stage of winter training, the proportion of each sports team has not been greatly adjusted. In 2020, the reaction stability index in the middle of winter training was significantly lower than that in the early stage of winter training, and it recovered in the later stage of winter training. This shows that in the middle stage of winter training, with the increase of simulated competition, the stimulation of athletes' load intensity on the pivot nerve is significantly higher than that in the early stage of winter training. Because the simulated competition is the actual competition of the whole team, this training method requires athletes' attention to be highly concentrated compared with ordinary tactical training. In the middle stage of winter training, the recovery mode of each team is mainly physical relaxation. The lack of recovery of the central nervous system may also be one of the reasons for the significant decline of the response stability index of the central nervous system at this stage.

## 5. Conclusion

1. The influence of different training loads on the results of real-time functional state test of female volleyball players is significant. Each index has different characteristics between the two experiments. Therefore, real-time functional state test plays a positive role in the diagnosis of fatigue of athletes' central nervous system.
2. HRV time-domain, frequency-domain, and central nervous system parameters are used to evaluate the physical function of elite women volleyball players in the process of intensive training and preparation before the competition. From the results, it can be seen that SDNN in time-domain index is related to the average reaction time of central nervous system.
3. HRV time-domain index SDNN and frequency-domain index LF n.u. have a significant impact on the average reaction time. The specific impact law needs to be further explored.

## Figures and Tables

**Table 1:** General situation of research objects.

Gender	Age (years)	Training years (years)	Master (person)	Level 1 (people)
Female	21.3 ± 3.1	7.9 ± 2.7	101	24

**Table 2:** List of test indicators.

	Test indicators					
Time-domain indicators	SDNN	RMSSD	SDSD	PNN50		
Frequency-domain index	LF/HF	HF	HF n.u.	LF	LF n.u.	VLF
Indicators of central nervous system	Quiet potential value	Average reaction time	Functional level index of sensory motor nervous system	Reaction stability index	Sensory motor development potential index	Tension index

**Table 3:** Changes of HRV time-domain indexes of women volleyball players at different load stages.

	SDNN	RMSSD	SDSD	PNN50
The early stage of the winter training	52.31 ± 2.24	62.12 ± 3.45	81.17 ± 4.12	18.96 ± 2.94
Middle of winter training	55.93 ± 2.64	66.22 ± 3.56	85.91 ± 4.95	21.86 ± 1.16
Late winter training	69.67 ± 3.69	81.34 ± 4.43	103.36 ± 5.91	22.54 ± 1 .22
*F*	2.268	1.414	1.126	0.574
*P*	0.114	0.252	0.332	0.568

**Table 4:** Changes of HRV frequency-domain indexes of female volleyball players at different load stages.

	LF/HF	HF	HF n.u.	LF	LF n.u.	VLF
The early stage of the winter training	0.85 ± 1.45	836.46 ± 32.57	69.67 ± 2.72	293.64 ± 30.67	30.33 ± 2.72	92.59 ± 3.78
Middle of winter training	0.42 ± 0.30	746.62 ± 35.09	73.38 ± 3.40	293.58 ± 30.55	26.62 ± 3.40	90.79 ± 3.16
Late winter training	0.99 ± 2.18	1300.62 ± 37.56	67.49 ± 1.62	989.18 ± 12.46	32.51 ± 9.62	101.69 ± 6.74
*F*	0.770	1.628	0.492	2.394	0.492	0.134
*P*	0.468	0.205	0.614	0.100	0.614	0.875

**Table 5:** Changes of central nervous system indexes of female volleyball players at different loading stages.

	A	B	C	D
The early stage of the winter training	−1.03 ± 0.016	4.79 ± 0.21	3.78 ± 0.42	117.31 ± 3.5
Middle of winter training	0.64 ± 0.042	4.64 ± 0.21	3.42 ± 0.49	93.50 ± 1.94
Late winter training	−0.52 ± 0.09	4.72 ± 0.27	3.66 ± 0.52	118.43 ± 7.07
*F*	0.075	2.627	3.757	0.517
*P*	0.927	0.079	0.026	0.597

## Data Availability

The data used to support the findings of this study have not been made available because they are confidential.
